# High-density lipoprotein cholesterol efflux capacity in patients with obstructive sleep apnea and its relation with disease severity

**DOI:** 10.1186/s12944-022-01723-w

**Published:** 2022-11-07

**Authors:** Reza Fadaei, Samaneh Mohassel Azadi, Eric Rhéaume, Habibolah Khazaie

**Affiliations:** 1 Sleep Disorders Research Center, Kermanshah University of Medical Sciences, Kermanshah, Iran; 2 Department of Clinical Biochemistry, Faculty of Medicine Tehran University of Medical Sciences, Tehran, Iran; 3 Montreal Heart Institute, 5000 Belanger Street, Montreal, H1T 1C8 Canada; 4 Department of medicine, Université de Montréal, 2900 Edouard-Montpetit boulevard, Montreal, H3T 1J4 Canada

**Keywords:** HDL function, Anti-oxidant, Apolipoprotein, Dyslipidemia, Cholesterol efflux, Apnea

## Abstract

**Background:**

Obstructive sleep apnea (OSA) is linked to an accelerated risk of cardiovascular disease (CVD). Some key CVD risk factors are present in patients suffering from OSA such as hypertension, inflammation, oxidative stress, and dyslipidemia. High-density lipoprotein (HDL) cholesterol efflux capacity (CEC) is proposed as a reliable biomarker of HDL function and the present study aimed to quantify this biomarker in patients with OSA.

**Methods:**

ATP binding cassette subfamily A member 1 (ABCA1), non-ABCA1, and total CEC were determined in 69 polysomnographic-confirmed OSA patients and 23 controls. Moreover, paraoxonase (PON) activities, high-sensitivity C-reactive protein (hsCRP), apolipoprotein B (apo B), and apolipoprotein A-I (apo A-I) circulating levels were quantified in the studied population.

Results: All CEC measures were reduced in the OSA group compared to the control group. Strikingly, ABCA1 CEC was diminished in severe OSA in comparison with mild OSA. Furthermore, PON activities and apo A-I showed lower levels, while hsCRP and apo B were elevated in OSA patients compared to controls. Moreover, ABCA1 CEC showed an inverse association with hsCRP and a positive association with apo A-I, while non-ABCA1 CEC presented an association with HDL-C.

**Conclusion:**

These results suggest the presence of an impaired HDL function in OSA. In particular, ABCA1 CEC was associated with disease severity and inflammation which could be a factor increasing the risk of CVD.

**Supplementary Information:**

The online version contains supplementary material available at 10.1186/s12944-022-01723-w.

## Introduction

Obstructive sleep apnea (OSA) is considered one of the most frequent sleep-related disorders and is stated to affect a large fraction of adults, mainly middle-aged men, and exceed more than 50% in some countries [1, 2]. Recurrent episodes of complete or incomplete upper airway collapse during sleep are the reasons for the disorder leading to a complete or near-complete cessation of breathing [3]. There are various risk factors suggested for the current increase in OSA incidence, including age, sex, and obesity [4]. In addition to sleep-related manifestations of OSA, such as excessive sleepiness during the daytime, associations between OSA and cardiovascular diseases (CVD) and atherosclerotic CVD (ASCVD) were reported in previous research [5–7]. Several lines of evidence showed that OSA promotes oxidative stress, vascular inflammation, dyslipidemia, metabolic syndrome, high blood pressure, and endothelial dysfunction which are known key risk factors for ASCVD incidence [8, 9].

Atherogenic dyslipidemia is mostly defined as the increase in the circulating low-density lipoprotein-cholesterol (LDL-C), total cholesterol (TC), and triglycerides (TG) and a decline in the levels of high-density lipoprotein-cholesterol (HDL-C) [10]. HDL plays a central role in promoting the efflux of cholesterol to the liver from peripheral tissues, which is probably its main antiatherogenic role [11]. Antioxidant, anti-inflammatory, and endothelial cell maintenance functions are other anti-atherosclerotic functions of HDL [12]. Antioxidant activity is one of the crucial activities of HDL particles to prevent atherosclerotic plaque formation and development [13]. Based on numerous studies, HDL’s antioxidant activity is involved in its potential to impair low-density lipoprotein (LDL) oxidation. It has been reported that various HDL protein components such as apolipoprotein A-I (apo A-I) and related hydrolases such as paraoxonase 1 (PON1) influence HDL’s antioxidant activity. PON1 activity is measured by paraoxonase and arylesterase activities and it has been shown that PON1 is the main factor that protects lipoproteins from oxidative stress and lipid peroxidation [14, 15].

Traditionally, HDL particles have been reported as the protective agent regarding CVDs [12, 16–18]. However, recent interventional and clinical studies suggested that the HDL-C level is an inadequate index to predict ASCVD [12]; hence cholesterol efflux capacity (CEC), a critical measurement of HDL function, is proposed as a more reliable marker for estimating the risk of ASCVD [19]. Indeed, recent studies have reported an independent relation between cholesterol efflux from macrophages and ASCVD [20, 21].

Efflux of cholesterol from peripheral tissues is the first step in the reverse cholesterol transport (RCT) pathway, and the potential of HDL particles to promote cholesterol efflux from donor cells such as macrophages is measured by CEC [22]. HDL functions, in particular CEC, are influenced by several factors such as the lipid and protein composition of HDL, inflammatory conditions, and oxidative stress [23]. It has been demonstrated that inflammation and oxidative stress are elevated in patients suffering from OSA [24, 25]. Inflammation and pro-inflammatory mediators can suppress apo A-I expression in the liver, which leads to a decrease in HDL levels [26]. In addition, during inflammation, serum amyloid A (SAA) level is increased and can replace apo A-I in HDL leading to rapid clearance from the circulation, which in turn contributes to the further decrease of HDL and apo A-I levels [27, 28].

Impaired HDL CEC was reported in patients suffering inflammatory and cardiometabolic diseases [29–31]. However, no data have been reported on HDL CEC in patients suffering from OSA. Therefore, the current study sought to investigate the change in HDL CECs and their associations with OSA parameters and biochemical measurements.

## Materials and methods

### Study population

This case-control study included 92 subjects who performed polysomnography (PSG) tests: 69 patients with OSA, and 23 controls. To normalize data and eliminate bias, all subjects with a history or evidence of kidney disease, diabetes, cardiovascular disease, inflammatory diseases such as systemic lupus erythematosus and rheumatoid arthritis, alcohol consumption, and using lipids and/or cholesterol-lowering agents, and anti-inflammatory medication during the last 3 months were excluded from the study. Moreover, patients were treatment-naive and received no treatment for their sleep before the study and controls had normal PSG result and were from any sleep disorder. The Ethics Committee of the Kermanshah University of Medical Sciences approved the study (ethic code: IR.KUMS.REC.1398.685). An informed written consent form was signed by all subjects according to the “Declaration of Helsinki”.

### Polysomnography

All participants underwent an overnight polysomnography test using SOMNOscreen™ plus (SOMNOmedics GmbH, Randersacker, Germany) as previously described [32, 33]. The continuous PSG recordings were performed for 7 hrs, as previously described [34]. The American Academy of Sleep Medicine (AASM 2012) guidelines were applied to define sleep-related parameters. Accordingly, hypopnea is considered as reduced airflow by ≥30% accompanied by a decline in oxygen desaturation index of ≥3% or arousal, whereas a complete cessation of breathing (lasting for ≥10 seconds) is considered apnea. The apnea-hypopnea index (AHI) is defined as the mean number of hypopneas and apneas per hour.

The criteria for OSA diagnosis were an AHI score of AHI ≥ 5 events/h, and controls had AHI < 5. Moreover, patients were classified into three subgroups according to their AHI scores: 1) Mild-OSA (*n* = 24): 5 ≤ AHI < 15, 2) Moderate-OSA (*n* = 21): 15 ≤ AHI < 30, and 3) Severe-OSA (*n* = 24): AHI ≥ 30 [35].

### Anthropometric and laboratory assessment

A standard sphygmomanometer was employed to evaluate blood pressure, and it was performed in a sitting position after 15 minutes of rest. The equation of [height (m)/weight (kg)] has been used to calculate the BMI of each subject. The venous blood samples were taken from all participants after one night of fasting, and the serum was immediately prepared. An auto-analyzer, using commercially available kits (ParsAzmoon, Iran) was employed to assess the fasting blood glucose (FBG) and lipid profiles, including triglyceride (TG), total cholesterol (TC), low-density lipoprotein-cholesterol (LDL-C), and high-density lipoprotein-cholesterol (HDL-C). The atherogenic index of plasma was calculated by the following formula: log (TG/HDL-C). An aliquot of serum was stored at − 70 °C for future analyses.

### Apolipoproteins and enzyme activity measurements

ELISA kits (Abcam, USA) were used to investigate the serum level of apoA-I and apolipoprotein B (apoB). The intra and inter-assay coefficients of variation (CV), and for apoB measurement were 6.5 and 6.6%, and for apoA-I were 5.6 and 6.3%, respectively. High-sensitivity C-reactive protein (hsCRP) was evaluated by an ELISA kit (Monobind, USA) with intra and inter CV of 5.4 and 4.8%, respectively.

Paraoxonase and arylesterase activities were determined as previously described [36]. Briefly, paraoxon substrate was used to determine paraoxonase activity, and phenylacetate was the substrate to quantify arylesterase activity. P-nitrophenol is produced as the result of paraoxonase activity and its absorbance was determined using a plate reader at 405 nm. In addition, arylesterase activity produces phenylacetate and its absorbance was measured at 270 nm.

### Cholesterol efflux capacity

The apoB-depleted serum has been used for determining the CEC. The polyethylene glycol (PEG) precipitation method was employed to prepare apoB-depleted serum. Briefly, samples were mixed with PEG 6000 in 10 mM HEPES, pH 8.0, and centrifuged for 30 min at 2200 g (at 4C°) [37].

Three CEC measures were investigated in all subjects: 1) ATP-binding cassette transporter1 (ABCA1)-mediated CEC pathway, 2) non-ABCA1 CEC pathway, and 3) total CEC. J774 murine macrophages were obtained from the cell bank of the Pasteur Institute, Iran. J774 murine macrophages were cultured in RPMI-1640 medium enriched with 10% of foetal bovine serum and labelled using ^3^H-cholesterol (1 μCi) (Perkin Elmer, USA). After a 24-hour incubation period, the medium was discarded, and PBS was used to wash the cells. From here, the cells were split into two groups and cultured for 6 h, one in the presence of 8-(4-chlorophenylthio)-cAMP (Sigma, USA), a potent stimulator to upregulate ABCA1 expression in J774 cells, and the other in its absence. In the next step, the medium was supplemented with apoB-depleted serum (2.8% final concentration) and incubated for 4 h. Finally, liquid scintillation counting was used to quantify the amount of ^3^H-cholesterol in cell lysate and medium. In all steps, 2 μg/mL of Acyl-CoA cholesterol acyltransferase (ACAT) inhibitor (Sandoz 58,035, Sigma, USA) was used. Total CEC, non-ABCA1 CEC, and ABCA1 CEC were defined as the CEC obtained in cAMP-treated cells, CEC measured in cAMP-free cells, and the difference between total and non-ABCA1 CEC, respectively [38].

The following formula was applied to calculate the CEC: [radioactivity in efflux medium/total radioactivity in medium and cells) × 100]. The efflux in the plate without apoB-depleted serum was subtracted from efflux induced by the apoB-depleted serum medium. Each efflux analysis was performed in duplicates to validate data. Furthermore, the results of each experiment were normalized to the results of the standard, which was a pooled sample of ten controls. The intra-assay CV for total CEC and non-ABCA1 CEC were 6.8 and 7.2%, while the inter-assay was 6.5 and 7.1%, respectively.

### Statistical analysis

A Chi-square test was applied to report the categorical data as frequency and percentage. The normality of continuous data was examined by the Kolmogorov–Smirnov test. The student’s t-test and one-way analysis of variance (ANOVA) were used to analyse normal-distributed data and reported by the mean and standard deviation (SD). Mann-Whitney U test and Kruskal Wallis were used to analyse skewed data; the results were shown as the median and interquartile range (IQR) and were logarithmically converted before correlation and regression analysis. To explore the correlation between CECs and continuous variables, Spearman correlation tests were carried out. Correlated factors with CECs were included in multiple linear regression models. To analyse the obtained data, SPSS software version 18 was used, and a statistically significant threshold was considered as a two-sided *p*-value less than 0.05. The sample size was calculated according to a previous study [30] with a power of 80% and a *p*-value of 0.05 in total CEC between OSA and control groups.

## Results

### Basic characteristics of the studied populations

The findings of anthropometric and biochemical measures are given in Table 1. Age, sex, and BMI displayed no significant change between OSA patients and controls. As predicted, AHI, AHI of rapid eye movement (AHI_REM_) stage and AHI of non-rapid eye movement (AHI_NREM_) stage were considerably higher in patients suffering OSA compared to controls, although average SpO2 was not considerably different in OSA patients compared to controls. FBG revealed no significant difference between the two groups, while insulin and HOMA-IR were observed to be greater in OSA patients than in controls. TG levels and AIP were elevated in patients with OSA, and there was a trend for HDL-C to be lower in OSA patients however it did not reach the significant threshold (*P* = 0.05). LDL-C and TC showed no remarkable change between the groups.

**Table 1:** Basic characteristics of the studied population

Variable	Control (***n*** = 23)	OSA (***n*** = 69)	***P***-value
Sex (Male)	14 (60.9%)	50 (72.5%)	0.247
Age (Year)	46.2 ± 9.8	46.5 ± 12.6	0.916
BMI (kg/m^2^)	26.96 ± 3.56	26.82 ± 2.53	0.861
SBP (mmHg)	116.1 ± 10.2	119.8 ± 16.8	0.335
DBP (mmHg)	72.3 ± 6.9	76.5 ± 7.1	0.016
AHI (events/h)	2.39 ± 1.06	25.34 ± 19.45	< 0.001
AHI_REM_ (events/h)	1.01 ± 0.71	15.72 ± 11.86	< 0.001
AHI_NREM_ (events/h)	2.89 ± 1.25	38.86 ± 25.67	< 0.001
Average SpO2 (%)	92.82 ± 4.68	91.69 ± 4.65	0.316
FBG (mg/dL)	93.63 ± 13.31	96.28 ± 11.23	0.353
Insulin (μU/mL)	3.39 ± 1.78	5.53 ± 3.91	0.001
HOMA-IR	0.79 ± 0.42	1.32 ± 0.94	< 0.001
TC (mg/dL)	153.4 ± 31.9	165.6 ± 43.1	0.216
TG (mg/dL)	113.1 ± 40.2	142.9 ± 52.5	0.015
HDL-C (mg/dL)	46.98 ± 7.28	42.64 ± 9.58	0.050
LDL-C (mg/dL)	92.0 ± 26.5	100.6 ± 31.5	0.240
AIP	0.361 ± 0.191	0.508 ± 0.12	0.003

*BMI* Body mass index, *SBP* Systolic blood pressure, *DBP* Diastolic blood pressure, *AHI* Apnea hypopnea index, *REM* Rapid eye movement, *NREM* Non-rapid eye movement, *FBG* Fasting blood glucose, *HOMA-IR* Homeostatic model assessment for insulin resistance, *TC* Total cholesterol, *TG* Triglyceride, *HDL-C* High density lipoprotein-cholesterol, *LDL-C* Low density lipoprotein-cholesterol, *AIP* Atherogenic index of plasma

### Apolipoproteins, paraoxonase activities

**Fig. 1:**
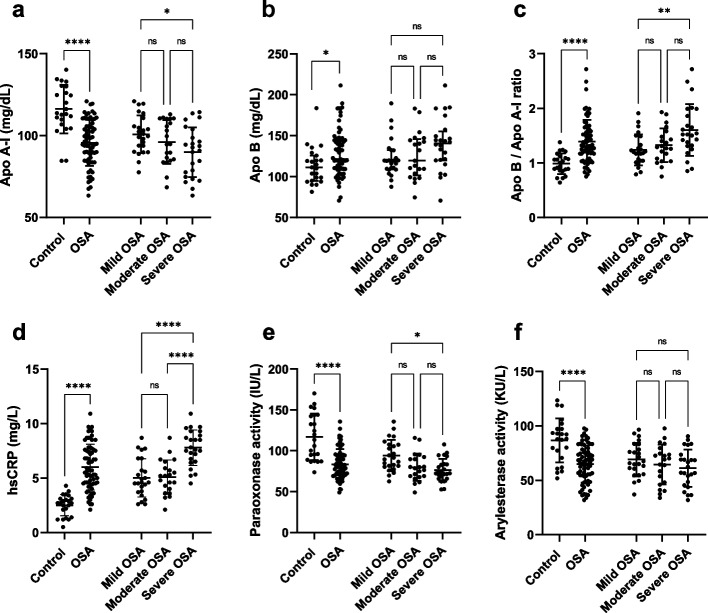
Serum levels of apolipoproteins and HDL-related enzyme activities. **a** Apo A-I was lower in OSA group in comparison to the controls, in addition, circulating apo A-I were lower in patients with severe OSA compared with patients with mild OSA. **b** Apo B indicated a higher concentration in OSA patients compared with control individuals, while there was no considerable difference between categories of OSA. **c** The apo B / apo A-I ratio is elevated in OSA patients in comparison to controls, and severe OSA groups displayed a higher apo B / apo A-I ratio compared to the mild OSA group. **d** HsCRP represented a higher concentration in patients suffering OSA compared to controls and severe OSA patients had higher levels of hsCRP compared to patients with mild and moderate OSA. **e** Paraoxonase activity was displayed a lower level in patients suffering OSA compared to controls, and severe OSA group showed lower activity of paraoxonase compared to mild OSA group. **f** Patients with OSA demonstrated lower arylesterase activity compared to controls, and there was no remarkable change between the OSA categories

Apo A-I was significantly decreased in the patients (95.5 ± 14.1 mg/dL) in comparison to the controls (116.2 ± 14.8 mg/dL, *P* < 0.001), and there was no remarkable change in apo A-I levels between the OSA categories (Fig. 1a). Apo B was increased in the patients (121.4 (109.3, 145.5) mg/dL) compared to the controls (111.2 (94.86, 125.7) mg/dL, *P* = 0.017), however, there was no significant change in apo B levels between the OSA categories (Fig. 1b). Moreover, the apo B/apo A-I ratio was higher in patients (1.39 ± 0.40) than in controls (0.99 ± 0.19, *P* < 0.001), and severe OSA patients (1.60 ± 0.47) had a higher apo B/apo A-I ratio than the mild OSA group (1.24 ± 0.28, *P* = 0.041) (Fig. 1c).

HsCRP was significantly increased in OSA patients (6.0 ± 2.1 mg/L) compared with controls (2.51 ± 0.98 mg/L, *P* < 0.001), in addition, severe OSA patients (7.8 ± 1.6 mg/L) had higher hsCRP concentration compared to mild (5.0 ± 1.8 mg/L, *P* < 0.001) and moderate OSA (5.1 ± 1.6 mg/L, *P* < 0.001) (Fig. 1d). Paraoxonase activity (117 ± 28.4 vs. 83.7 ± 18.2 IU/L, *P* < 0.001) was found to be lower in OSA patients compared to controls and it also showed lower activity in the severe OSA group (13.9 ± 2.8 IU/L,) compared to the mild group (19.0 ± 3.9 IU/L, *P* = 0.001) (Fig. 1e). Arylesterase activity (86.7 ± 20.3 vs 64.9 ± 17.0 KU/L, *P* < 0.001) was elevated in OSA patients compared to controls, while arylesterase activity showed no significant difference between OSA categories (Fig. 1f).

### Cholesterol efflux capacity

**Fig. 2:**
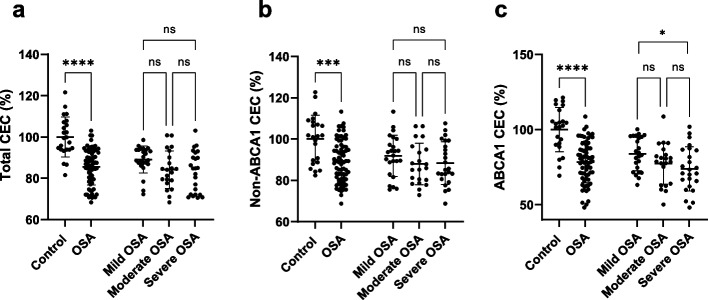
Cholesterol efflux capacities. **a** Total CEC is declined in OSA in comparison to control individuals, and there was no change between OSA categories. **b** non-ABCA1 CEC illustrated a significant decline in OSA patients compared with controls and it showed no remarkable change between categories of OSA. **c** ABCA1-dependent CEC demonstrated a lower value in OSA patients compared with controls and severe OSA patients indicated lower ABCA1 CEC compared to the mild OSA group

As shown in Fig. 2a, total CEC was found to be lower in OSA patients (85.6 ± 8.9) compared to controls (100 ± 9.6, *P* < 0.001), and the difference between the groups remained significant after adjustment for age, sex, BMI and HOMA-IR (99.5 ± 9.5 vs. 85.7 ± 5.4, *P* < 0.001), whereas there was no remarkable change between OSA categories. Similarly, non-ABCA1 CEC represented a lower level in OSA patients (89.5 ± 10.2) compared to the control group (100 ± 11.6, *P* < 0.001, Fig. 2b)) and adjustment for age, sex, BMI, and HOMA-IR had no impact on the difference between the groups (99.7 ± 11.1 vs. 89.6 ± 6.3, *P* < 0.001), however, OSA categories showed no significant change. In addition, ABCA1-dependent CECs were found to be diminished in OSA patients (78.4 ± 13.7) compared to controls (100 ± 14.7, *P* < 0.001, Fig. 2e), and the results remained unchanged after adjustment for age, sex, BMI and HOMA-IR (99.0 ± 14.1 vs. 78.8 ± 8.0, *P* < 0.001). In addition, ABCA1-dependent CECs showed higher levels in mild OSA patients (83.9 ± 11.2) compared to severe OSA patients (73.9 ± 14.7, *P* = 0.033, Fig. 2c).

Furthermore, the odds ratio of OSA based on changes in CECs was determined using binary logistic regression, and the findings are displayed in Table 2. All three categories of CEC were associated with OSA. In addition, adjustments were performed for possible covariates (age, sex, and BMI) and all associations remained significant.

**Table 2:** The odd ratio of OSA according to one present change in CECs

Models	Odd ratio	95% CI	***P***-value
Total CEC			
Crude	0.817	0.742, 0.899	< 0.001
^a^Adjusted	0.814	0.737, 0.899	< 0.001
Non-ABCA1 CEC			
Crude	0.913	0.867, 0.961	< 0.001
^a^Adjusted	0.913	0.866, 0.962	< 0.001
ABCA1 CEC			
Crude	0.893	0.848, 0.940	< 0.001
^a^Adjusted	0.881	0.831, 0.933	< 0.001

^a^An adjustment was performed for age, sex, and body mass index

*CEC* Cholesterol efflux capacity

### Association of cholesterol efflux capacities with other variables

Results of CECs were analysed according to sex to find possible relations of CECs with sex. All three CEC measurements revealed no statistically significant change between females and males in the whole population and also in controls and OSA separately (S Fig. 1). Furthermore, the studied population was divided according to BMI (cut off: 25) and there was no difference between subjects in normal weight (BMI < 25) and overweight (BMI > 25) in the whole population and OSA and control separately (S Fig. 2).

A correlation analysis was performed on controls and patients separately, and the detailed results are displayed in Table 3. In control individuals, non-ABCA1 CEC was directly correlated with HDL-C. In the OSA group, total CEC inversely correlated with AHI, TG, AIP, apo B/apo A-I and positively correlated with apo A-I and HDL-C. In addition, stepwise multiple linear regression revealed that apo A-I (B (95%CI): 0.229 (0.090, 0.368), *P* = 0.002) and TG (B (95%CI): − 0.043 (− 0.08, − 0.005), *P* = 0.026) were the main predictors of total CEC. ABCA1 CEC showed an inverse correlation with AHI, TG, LDL-C, hs-CRP and apoB/apo A-I ratio, and positively correlated with apo A-I, and stepwise multiple linear regression indicated that apo A-I (B (95%CI): 0.297 (0.075, 0.518), *P* = 0.009) and hsCRP (B (95%CI): − 2.044 (− 3.529, − 0.559), *P* = 0.008) were the strongest predictors of ABCA1 CEC. Moreover, non-ABCA1 CEC indicated a positive correlation with HDL-C and apo A-I and was independently associated with HDL-C (B (95%CI): 0.541 (0.252, 0.830), *P* < 0.001).

**Table 3:** Correlation of cholesterol efflux capacities (CEC) with other variables

	Control			OSA		
	Total CEC	ABCA1 CEC	Non ABCA1 CEC	Total CEC	ABCA1 CEC	Non ABCA1 CEC
Age	0.035	−0.260	0.226	−0.071	− 0.137	0.005
BMI	−0.060	− 0.003	− 0.075	−0.126	− 0.164	−0.049
SBP	0.002	0.024	−0.014	−0.097	− 0.193	0.012
DBP	−0.088	−0.075	− 0.061	−0.033	− 0.058	−0.002
AHI	−0.079	0.273	−0.292	−0.347**	− 0.353**	−0.208
AHI_REM_	−0.082	0.188	−0.255	−0.286*	− 0.316*	−0.204
AHI_NREM_	−0.103	0.275	−0.277	−0.389**	− 0.397**	−0.251
AverageSpO2	0.235	0.167	0.186	0.108	0.206	−0.006
FBS	−0.023	0.027	−0.048	−0.018	− 0.222	0.140
Insulin	−0.108	−0.124	− 0.053	−0.160	− 0.173	−0.089
HOMA-IR	−0.091	−0.102	− 0.045	−0.173	− 0.221	−0.070
TC	0.178	0.131	0.137	−0.113	−0.205	− 0.002
TG	0.028	0.139	−0.061	−0.333**	− 0.296*	−0.231
HDL-C	0.400	0.097	0.447^*^	0.378**	0.129	0.415**
LDL-C	0.133	−0.235	0.334	−0.160	− 0.305*	0.009
AIP	−0.281	−0.291	− 0.097	−0.476**	− 0.411**	−0.314**
Apo A-I	0.397	0.359	0.260	0.419**	0.417**	0.258*
Apo B	0.094	−0.121	0.205	−0.112	− 0.121	−0.062
ApoB/ApoA-I	−0.180	−0.390	0.041	−0.350**	− 0.342**	−0.219
hsCRP	−0.008	0.093	−0.183	−0.359**	− 0.422**	−0.173
Arylesterase	0.190	−0.047	0.277	0.136	−0.012	0.192
Paraoxonase	0.141	0.011	0.174	0.155	0.106	0.131

*BMI* Body mass index, *SBP* Systolic blood pressure, *DBP* Diastolic blood pressure, *AHI* Apnea hypopnea index, *REM* Rapid eye movement, *NREM* Non-rapid eye movement, *FBG* Fasting blood glucose, *HOMA-IR* Homeostatic model assessment for insulin resistance, *TC* Total cholesterol, *TG* Triglyceride, *HDL-C* High density lipoprotein-cholesterol, *LDL-C* Low density lipoprotein-cholesterol, *AIP* Atherogenic index of plasma, *apo A-I* Apolipoprotein A-I, *apo B* Apolipoprotein B, *hsCRP* High sensitivity C-reactive protein

## Discussion

OSA is the most prevalent sleep disorder and has been reported to be linked with inflammation, oxidative stress as well as atherogenic dyslipidemia in these patients [39, 40]. Furthermore, it has been demonstrated that the amount of HDL-C in OSA patients is lowered [41]. According to recent studies, HDL-C levels did not represent a strong marker to predict ASCVD; hence HDL function such as CEC was suggested as a more informative index for ASCVD prediction [19]. In the present study, the CEC index has been explored in patients with OSA, for the first time.

All of the CEC measures (total, non-ABCA1, and ABCA1-mediated CECs) were reduced in the OSA group compared to the control group. The presents study substantially extends a smaller earlier study of 12 patients and 6 controls by Xu et al., which demonstrated reduced patients’ peripheral blood mononuclear cell (PBMC)-derived macrophage ability to efflux cholesterol through ABCA1, downregulation of the ABCA1 gene in macrophages of patients with OSA, and an inverse correlation of AHI with macrophage ABCA1 CEC and expression [42]. It is worthy to note that the findings of the present study and the study by Xu et al., indicated that two important contributors to cholesterol efflux (HDL as acceptor and ABCA1 as the most important transporter) are impaired in OSA patients. OSA has a close relation with obesity and metabolic syndrome and previous research found lower HDL CEC in individuals with cardiometabolic disorders and obesity [23, 30, 43, 44]. It should be mentioned that the groups have been matched in terms of BMI and the results showed that decreased HDL CECs were independent of BMI. Notably, the current study’s findings demonstrated a negative relation between ABCA1-mediated CEC and AHI and disease severity. Moreover, there is evidence that AHI_non-REM_ has a stronger relation with dyslipidemia in patients with OSA [45, 46] and in line with this concept, our correlation analysis indicated a stronger correlation of total and ABCA1 CECs with non-REM AHI in comparison to AHI_REM_. On the one hand, it has been demonstrated that OSA severity is associated with consequences of the disease such as CVD death, oxidative stress, and inflammation [47, 48]. In line with this concept, Peker et al. have investigated AHI’s impact on death rates in people with coronary heart disease; their results have indicated that the death rate in the studied population was remarkably greater in OSA patients with AHI higher than 10 [49]. Another study conducted by Turmel et al. reported that the area of coronary plaque in patients with AHI greater than 15 is considerably larger than in individuals with AHI less than 15 [50]. On the other hand, ABCA1-mediated CEC has been proposed as one of the most important CEC pathways to protect against ASCVD [51]. It is well known that ABCA1 CEC is dependent on apo A-I and nascent HDL [51]. In this regard, as the first step in RCT, phospholipids and free cholesterol are exported from cells through the interaction apo A-I with ABCA1 [52]. In line with this fact, the findings of the present study showed that the apo A-I level has an independent association with ABCA1-mediated CEC in OSA patients. In addition, hsCRP showed a close inverse relation with ABCA1 CEC. Evidence shows that inflammation suppresses RCT through multiple pathways. Inflammation upregulates miR-33 which in turn reduces ABCA1 and ABCG1 expression, moreover, inflammation leads to suppression of apo A-I expression in hepatocytes [53]. Since inflammation has the ability to alter RCT, HDL function, and apo A-I, the current study’s findings propose that inflammation might be the main underlying mechanism for the CEC reduction in patients with OSA [54]. In addition, all CECs presented inverse correlations with AIP which was reported to be higher in patients with OSA [55, 56]. These findings suggest that TG/cholesterol ester exchange between HDL and other lipoproteins might be another factor that affected HDL function.

The findings indicate that HDL-C had positive correlations with total and non-ABCA1-CEC. It has been well established that HDL is the main acceptor of cholesterol efflux through ABCG1 and SR-B1 [51]. This is most likely because HDL is phospholipid containing and both ABCG1 and SR-B1 interact with lipidated lipoprotein particles. This differs from ABCA1 which preferentially interacts with lipid-poor apoA-I-containing particles.

In addition to CECs, the findings demonstrated an impaired anti-oxidant function of HDL, measured by PON1 activities (paraoxonase and arylesterase), in patients with OSA. According to previous studies, HDL’s antioxidant properties help to prevent coronary heart disease [11]. PON1 activities suppress the oxidation of LDL which is the initiator of atherosclerosis in the arterial intima [5]. In line with the present study, Tan et al. showed that the HDL’s anti-oxidant activity index is reduced significantly in patients with OSA than in controls [57]. Lavie et al. showed lower PON1 in OSA. They investigated the concentration of lipid peroxidation biomarkers in patients with OSA and reported decreased PON1 activity in OSA subjects compared to controls and similar results between severe and moderate OSA [39]. However, a meta-analysis demonstrated no significant change in PON1 and arylesterase activities in OSA patients [58].

### Comparisons with other studies and what does the current work add to the existing knowledge

For the first time, the findings of the present study demonstrated impaired CECs in patients suffering OSA compared to controls, which extended the existing results of OSA patients PBMC-derived macrophages’ suppressed ability to efflux cholesterol through ABCA1 and the downregulation of macrophages’ ABCA1 gene expression [42].

### Strengths and limitations

The present study was performed on a matched population in terms of sex, age, and BMI, and the diagnosis was based on polysomnography as the gold standard of OSA diagnosis. There are limitations to the present study: there are no data on subclinical atherosclerosis such as carotid intima-media thickness which limited us in ouranalysis of the relation of CECs with atherosclerosis in OSA patients. Determining the size and subclass HDL could be useful data related to HDL function but these have not been determined here. Moreover, this is a cross-sectional study, therefore causality cannot be concluded. Furthermore, the impact of treatment (i.e. CPAP) was not evaluated and there were no data on diet.

## Conclusion

Taken together, this study found that the CEC function of HDL is reduced in patients with OSA independent of sex and BMI. The CEC decrease might be attributed to the significantly decreased circulating apo A-I levels and increased inflammation in patients with OSA. HDL’s antioxidant activity is also reduced among OSA subjects. It seems likely that multiple HDL functions and apo A-I levels may aid in the assessment of CVD risk in OSA patients. These findings showed one of the possible mechanisms for accelerating ASCVD in OSA patients and can be considered in the assessment of ACVD risk and preventing ASCVD in OSA patients. Furthermore, therapies to reduce inflammation and oxidative stress might be useful for improving the function of HDL in patients with OSA.

## Supplementary Information

**Additional file 1: S Figure 1.** Cholesterol efflux capacities according to sex. a) Total, b) non-ABCA1, and c) ABCA1 CEC displayed no statistically remarkable difference between females and males in the whole population. d) Total CEC, e) Non-ABCA1 CEC, and f) ABCA1 CEC indicated no remarkable difference in males compared to females in both OSA patients and controls.

**Additional file 2: S Figure 2.** Cholesterol efflux capacities according to BMI categories. a) Total, b) Non-ABCA1, and c) ABCA1 CEC have shown no considerable change between normal weight (BMI < 25) and overweight (BMI > 25) in the whole population. d) Total CEC, e) Non-ABCA1 CEC, and f) ABCA1 CEC demonstrated no change in normal weight (BMI < 25) compared with overweight (BMI > 25) in both OSA patients and controls.

## Data Availability

The data that support the findings of this study are available on request from the corresponding author (Habibolah Khazaie).
