# Supplementary material for: High-density lipoprotein cholesterol efflux capacity in patients with obstructive sleep apnea and its relation with disease severity
Source: Lipids Health Dis. 2022 Nov 7;21:116. doi: 10.1186/s12944-022-01723-w (PMC9639319; doi:10.1186/s12944-022-01723-w)
Supplement: Supplementary file 1 — Additional file 1: S Figure 1. Cholesterol efflux capacities according to sex. a) Total, b) non-ABCA1, and c) ABCA1 CEC displayed no statistically remarkable difference between females and males in the whole population. d) Total CEC, e) Non-ABCA1 CEC, and f) ABCA1 CEC indicated no remarkable difference in males compared to females in both OSA patients and controls. [file 12944_2022_1723_MOESM1_ESM.docx]

**S Figure 1. Cholesterol efflux capacities according to sex.** a) Total, b) non-ABCA1, and c) ABCA1 CEC displayed no statistically remarkable difference between females and males in the whole population. d) Total CEC, e) Non-ABCA1 CEC, and f) ABCA1 CEC indicated no remarkable difference in males compared to females in both OSA patients and controls.
