# Supplementary material for: High-density lipoprotein cholesterol efflux capacity in patients with obstructive sleep apnea and its relation with disease severity
Source: Lipids Health Dis. 2022 Nov 7;21:116. doi: 10.1186/s12944-022-01723-w (PMC9639319; doi:10.1186/s12944-022-01723-w)
Supplement: Supplementary file 2 — Additional file 2: S Figure 2. Cholesterol efflux capacities according to BMI categories. a) Total, b) Non-ABCA1, and c) ABCA1 CEC have shown no considerable change between normal weight (BMI < 25) and overweight (BMI > 25) in the whole population. d) Total CEC, e) Non-ABCA1 CEC, and f) ABCA1 CEC demonstrated no change in normal weight (BMI < 25) compared with overweight (BMI > 25) in both OSA patients and controls. [file 12944_2022_1723_MOESM2_ESM.docx]

**S Figure 2. Cholesterol efflux capacities according to BMI categories.** a) Total, b) Non-ABCA1, and c) ABCA1 CEC have shown no considerable change between normal weight (BMI<25) and overweight (BMI>25) in the whole population. d) Total CEC, e) Non-ABCA1 CEC, and f) ABCA1 CEC demonstrated no change in normal weight (BMI<25) compared with overweight (BMI>25) in both OSA patients and controls.
